# Functional microRNAs in Alzheimer’s disease and cancer: differential regulation of common mechanisms and pathways

**DOI:** 10.3389/fgene.2012.00323

**Published:** 2013-01-17

**Authors:** Kelly N. Holohan, Debomoy K. Lahiri, Bryan P. Schneider, Tatiana Foroud, Andrew J. Saykin

**Affiliations:** ^1^Department of Medical and Molecular Genetics, Indiana University School of MedicineIndianapolis, IN, USA; ^2^Training in Research for Behavioral Oncology and Cancer Control Program, Indiana University School of NursingIndianapolis, IN, USA; ^3^Center for Neuroimaging, Department of Radiology and Imaging Sciences, Indiana University School of MedicineIndianapolis, IN, USA; ^4^Department of Psychiatry, Indiana University School of MedicineIndianapolis, IN, USA; ^5^Melvin and Bren Simon Cancer Center, Indiana University School of MedicineIndianapolis, IN, USA

**Keywords:** microRNA, cancer, Alzheimer’s disease, pathways

## Abstract

Two of the main research priorities in the United States are cancer and neurodegenerative diseases, which are attributed to abnormal patterns of cellular behavior. MicroRNAs (miRNA) have been implicated as regulators of cellular metabolism, and thus are an active topic of investigation in both disease areas. There is presently a more extensive body of work on the role of miRNAs in cancer compared to neurodegenerative diseases, and therefore it may be useful to examine whether there is any concordance between the functional roles of miRNAs in these diseases. As a case study, the roles of miRNAs in Alzheimer’s disease (AD) and their functions in various cancers will be compared. A number of miRNA expression patterns are altered in individuals with AD compared with healthy older adults. Among these, some have also been shown to correlate with neuropathological changes including plaque and tangle accumulation, as well as expression levels of other molecules known to be involved in disease pathology. Importantly, these miRNAs have also been shown to have differential expression and or functional roles in various types of cancer. To examine possible intersections between miRNA functions in cancer and AD, we review the current literature on these miRNAs in cancer and AD, focusing on their roles in known biological pathways. We propose a pathway-driven model in which some molecular processes show an inverse relationship between cancer and neurodegenerative disease (e.g., proliferation and apoptosis) whereas others are more parallel in their activity (e.g., immune activation and inflammation). A critical review of these and other molecular mechanisms in cancer may shed light on the pathophysiology of AD, and highlight key areas for future research. Conclusions from this work may be extended to other neurodegenerative diseases for which some molecular pathways have been identified but which have not yet been extensively researched for miRNA involvement.

## INTRODUCTION

Cancer and neurodegenerative disease have become prominent areas of medical research in the United States, as these diseases afflict millions of Americans each year. Since age is a major risk factor for each disease area, our aging population makes progress in treatment a high priority (Qiu et al., 2009;Borgesius et al., 2011;Jeppesen et al., 2011;Howlader et al., 2012). Fundamental hallmarks of cancer include uncontrolled proliferation and disruption of apoptosis; conversely neurodegeneration is associated with increased cellular death (Hanahan and Weinberg, 2000;Leuner et al., 2012). Therefore, both diseases may potentially result from differential regulation of the same cellular pathways. Supporting this hypothesis, negative epidemiological correlations have been demonstrated between cancer and neurodegenerative diseases including Down’s syndrome, Parkinson’s disease (PD), Alzheimer’s disease (AD), schizophrenia, and multiple sclerosis (Tabares-Seisdedos et al., 2011). Interestingly, to date, although the rest of these neurodegenerative diseases appear to be associated with either increased or decreased comorbidity depending on the type of cancer, AD has been associated with a decreased co-occurrence of all types of cancer. A recent large-scale report indicated a significant negative correlation of cancer and AD in the Framingham Heart Study (Driver et al., 2012). In a longitudinal study, patients with AD also had a lower risk of developing cancer after adjusting for demographic factors (Roe et al., 2005). Further, Caucasian participants in another prospective study displayed a negative association between AD incidence and cancer risk (Roe et al., 2010).

**FIGURE 1:**
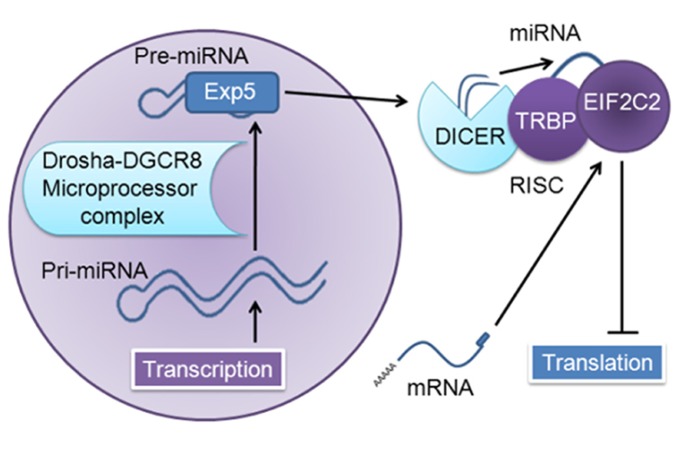
**MiRNA generation and function.** The nuclear transcript pri-miRNA is several kilobases in length; this transcript is cleaved by the microprocessor complex (DCGR8 and Drosha), which yields a short (~65 nucleotide) stem-loop pre-miRNA. This is transported out of the nucleus by Exportin 5 (Exp5) to the RNA-induced silencing complex (RISC) and cleaved by DICER1 (targeting the hairpin loop), generating a ~22 nucleotide miRNA duplex; one-strand is degraded, and the remaining strand is loaded into human immunodeficiency virus-1 transactivating response RNA-binding protein (TRBP)-recruited EIF2C2, also known as Argonaute 2, which then inhibits translation of target messenger RNAs (mRNAs;Bartel, 2004;Gregory et al., 2004, 2005;Chendrimada et al., 2005;Maniataki and Mourelatos, 2005;Kim et al., 2009;Rusca and Monticelli, 2011).

MicroRNAs (miRNAs) comprise one major post-transcriptional regulatory mechanism that has been implicated in a variety of cancers and neurodegenerative diseases (Zhang et al., 2007;Bushati and Cohen, 2008;Wu et al., 2008;Garzon et al., 2009a;Lau and de Strooper, 2010;Sonntag, 2010). As illustrated in **Figure 1**, miRNA is generated as a long precursor sequence in the nucleus, where it is cleaved to form a shorter stem-loop precursor, transported to the cytoplasm, and further processed in the RNA-induced silencing complex (RISC) by the ribonuclease DICER1 into a very short (~22 nucleotide) double-strand sequence; this is then unwound and one-strand is loaded onto EIF2C2, which consequently inhibits translation or results in cleavage of target messenger RNAs (mRNAs;Bartel, 2004;Gregory et al., 2004, 2005;Chendrimada et al., 2005;Maniataki and Mourelatos, 2005;Kim et al., 2009;Rusca and Monticelli, 2011).

The same miRNAs can be important to both types of disease; a few examples include miR-34b/c down-regulation in PD and small cell lung cancer, up-regulation of miR-206 as a protective effect against disease progression in a mouse model of amyotrophic lateral sclerosis and miR-206 down-regulation in laryngeal squamous cell carcinoma, and miR-132 down-regulation in frontotemporal dementia and methylation in prostate cancer (Williams et al., 2009;Minones-Moyano et al., 2011;Zhang et al., 2011b;Chen-Plotkin et al., 2012;Formosa et al., 2012;Tanaka et al., 2012). To date, there has been a focus on miRNAs in cancer. Although the roles of miRNAs in neurodegenerative diseases have not been as thoroughly investigated, these are currently under increasing scrutiny (Gascon and Gao, 2012). Given the plethora of information on cancer regulatory mechanisms including miRNA, as well as the possible involvement of some of the same miRNAs in cancer and neurodegeneration, consideration of the roles of miRNA functions in cancer might help elucidate corresponding or opposing functions in neurodegenerative disease. A more comprehensive review of the roles of miRNAs in cancer and neurodegeneration might yield insights into the underlying pathways involved in these diseases.

**FIGURE 2:**
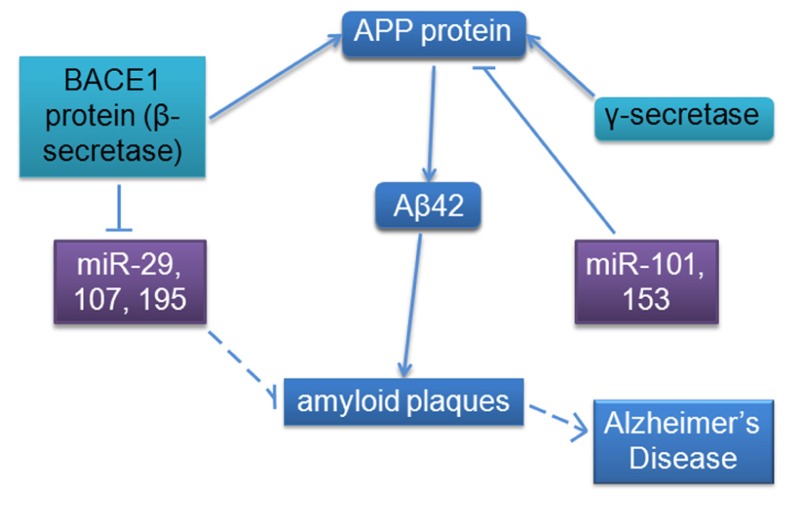
**MicroRNA involvement in the amyloid pathway appears to contribute to AD.** BACE1 mRNA expression appears to be redundantly regulated by multiple miRNAs (including predicted miR-9, not shown); APP mRNA expression is also regulated by miRNA. More miRNA binding sites have been bioinformatically predicted for both mRNAs, indicating that this regulatory mechanism is most likely very tightly regulated (Schonrock et al., 2012). Solid lines indicate known interactions; dashed lines indicate measured correlations.

The full spectrum of roles of miRNAs in different types of cancer and neurodegenerative diseases is a rich topic but beyond the scope of this targeted review; we decided to focus our analysis on select miRNAs implicated in both AD and cancer. AD pathology is characterized by an accumulation of extracellular amyloid plaques composed of amyloid-beta peptide fragment (Aβ) and intracellular neurofibrillary tangles composed of hyperphosphorylated protein tau, as well as neuronal loss in the hippocampus, temporal, and frontal lobes, increased inflammation, and oxidative stress (Glenner and Wong, 1984;Grundke-Iqbal et al., 1986;Kosik et al., 1986;Small and Duff, 2008;Gustaw-Rothenberg et al., 2010;Pavlides et al., 2010;Mohsenzadegan and Mirshafiey, 2012). We will review a number of studies linking miRNAs with differential expression and pathology in AD such as deposition of amyloid plaques and neurofibrillary tangles, as well as more specific pathway interactions and regulatory functions of the amyloid pathway, including regulation of amyloid protein precursor (APP) and beta-site APP cleaving enzyme 1 (BACE1 or β-secretase), a protease that cleaves APP to generate Aβ. The larger form of Aβ peptide containing 42 amino acid residues (Aβ42) mostly aggregates to form the previously mentioned amyloid plaques (see **Figure 2**).

We will focus on eight miRNAs (miR-9, -29a/b, -101, -107, -125b, -146a, -153, and -195) with differential expression in AD, evidence of correlation with disease pathology, and evidence of specific functional interactions, in order to investigate whether the large body of cancer research on these molecules may shed additional light on their function in AD, and whether it is also possible to use existing knowledge of these miRNAs in AD to further elucidate their functions in cancer. Serving as a case study of the potential utility of this type of cross-disease comparison, we suggest that miRNA research in cancer may lead to hypotheses for novel roles of these miRNAs in AD, and highlight important directions for future research.

We expect to observe miRNA involvement in cancer and AD in pathways that may be contributing to both pathologies, as well as activity in disease-specific pathways, and anticipate that the differential expression and or regulation of these miRNAs may contribute to the differences in disease pathology. In addition to the aforementioned proliferative/anti-apoptotic pathway, we expect to observe miRNAs involved in invasion, metastasis, inflammation, oxidative stress, and angiogenesis in cancer; since many of these pathways have also been implicated in neurodegeneration, we expect that these miRNAs will also be implicated in the same or related pathways in AD (Vagnucci and Li, 2003;Lukiw, 2004;Segal et al., 2004, 2005;Rojo et al., 2008;Maccioni et al., 2009;Streit et al., 2009;Tabares-Seisdedos et al., 2011;Cole and Sood, 2012;Vadnal et al., 2012).

**FIGURE 3:**
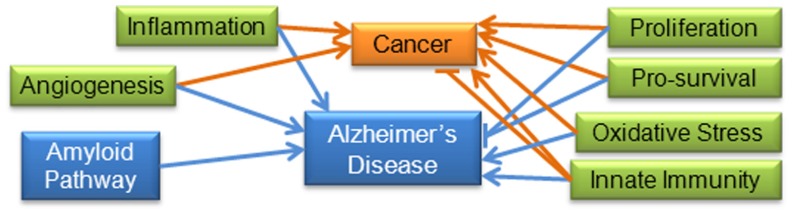
**Molecular and cellular pathways important to AD and cancer: increased levels of inflammation and oxidative stress have been positively associated with cancer and AD.** Angiogenesis is considered one of the hallmarks of cancer, and has also been shown to be triggered by amyloid in AD (Hanahan and Weinberg, 2000;Biron et al., 2011). Proliferation and pro-survival pathways have been shown to be positively associated with cancer and negatively associated with AD. Immunity has been shown to be both positively and negatively correlated with cancer (“immunoediting”), in addition to being positively correlated with AD. The amyloid pathway does not appear to be involved in cancer pathways, so for the purposes of this review is specific to AD. Blue lines indicate interactions found in AD research; orange lines indicate interactions found in cancer research.

For some pathways, including proliferation and pro-survival mechanisms, we expect to find evidence of inverse relationships (Driver and Lu, 2010;Driver et al., 2012;Hedskog et al., 2012); however, for inflammation, oxidative stress, and angiogenesis it is reasonable to expect similar activity since these are known factors in both areas of pathology (Ono, 2008;Rojo et al., 2008;Maccioni et al., 2009;Pavlides et al., 2010;Biron et al., 2011;Sethi et al., 2012;Vendramini-Costa and Carvalho, 2012). Although there is evidence that the neuro-immune and inflammatory pathways are up-regulated in AD, in cancer there is evidence that immunity can both suppress tumor growth and promote tumor progression, a process referred to as “immunoediting” (Maccioni et al., 2009;Schreiber et al., 2011;Vesely et al., 2011). **Figure 3** summarizes the documented and hypothesized roles of these pathways in cancer and AD. A comparison of miRNA expression and activity may yield more information on the contribution of these pathways to disease initiation and progression.

## miR-9

Three studies found that miR-9 was significantly up-regulated in the hippocampus and temporal lobe neocortex of AD brains compared to normal aging brains, indicating that miR-9 may play a pathological role in AD (Lukiw, 2007;Sethi and Lukiw, 2009;Lukiw et al., 2012). Because these brain regions show extensive pathology during the progression from health to AD (De Leon et al., 1997;Jack et al., 1998;Desikan et al., 2010;Nath et al., 2012;Spulber et al., 2012) up-regulation of miR-9 may play a role in degeneration. However, other studies found miR-9 was down-regulated in the hippocampus, anterior temporal cortex and medial frontal gyrus in AD patients (Cogswell et al., 2008;Hebert et al., 2008). Therefore, at present there is conflicting data regarding the specific role of miR-9 in AD, and its expression pattern in key cerebral regions and differences in sample characteristics and methods may be a factor in directionality of changes. It should be noted that miR-9 has a half-life of 30–60 min; the post-mortem interval (PMI) for the three studies finding up-regulation of miR-9 was 3 h or less, while the other two studies had PMIs of 3–10 and 24 h (Lukiw et al., 2012). It seems possible that this variability is enough to explain the measurement discrepancies, in which case miR-9 is up-regulated in AD brains, but decays at a rapid rate post-mortem, causing it to appear down-regulated at later time points.

In terms of mechanism, miR-9 has been predicted to target the 3′ untranslated region (UTR) of BACE1, a key element of the amyloid pathway, though to our knowledge there is currently no functional evidence of this interaction. If confirmed, this would support miR-9 down-regulation, presumably resulting in increased BACE1 expression activity and increased Aβ42 production (Hebert et al., 2008). Finally, there is some initial evidence that miR-9 may be up-regulated in response to interleukin-1B (IL-1β) and Aβ42 induced nuclear factor of kappa light polypeptide gene enhancer in B cells 1 (NF-κB), implying that this miRNA may be involved in the AD inflammatory and oxidative stress pathways (Lukiw, 2012). Brain-enriched miR-9 has also been implicated in a wide variety of functions in different life stages and organisms; overexpression in adult mouse neural progenitor cells has been shown to facilitate neuronal differentiation, although inhibiting miR-9 does not inhibit differentiation (Krichevsky et al., 2006;Yuva-Aydemir et al., 2011).

miR-9 is active in a variety of pathways, with both tumor suppressive and oncogenic functions for different types of cancer. Down-regulation has been observed in metastatic melanoma and head and neck squamous cell carcinoma (HNSCC;Liu et al., 2012b;Minor et al., 2012), while up-regulation has been observed in glioma, gastric cancer, biliary cancer, Hodgkin lymphoma (HL), colorectal cancer (CRC), breast cancer, and cervical cancer (Li et al., 2011b;Schraivogel et al., 2011;Shigehara et al., 2011;Inoue et al., 2012;Krell et al., 2012;Leucci et al., 2012;Wilting et al., 2012;Zhu et al., 2012b), indicating that this regulatory miRNA is likely involved in multiple pathways, some of which are differentially regulated in different cancer types.

There is functional evidence that this miRNA may act as a tumor suppressor in some cancers. Cell proliferation was significantly inhibited in a HNSCC cell line by overexpression of miR-9, indicating that it may be a negative regulator of this pathway (Minor et al., 2012). miR-9 was also shown to down-regulate proliferation and metastasis pathways in melanoma via the NF-κB1 molecular pathway; it was shown to directly target the NF-κB 3′ UTR. Since NF-κB1 is known to be activated by inflammatory and oxidative stress signals, this indicates that miR-9 may play an important role in these pathways in cancer as well as AD (Pavlides et al., 2010; ). miR-9 is up-regulated by NF-κB in AD, and down-regulates NF-κB in melanoma, suggesting that there may be a negative feedback loop in AD or differential regulation of this mechanism in AD and cancer. Additional research will be required to determine which of these explanations is correct.

In contrast to the role of miR-9 in suppressing proliferation and metastasis, and possible involvement in the NF-κB inflammatory and oxidative stress pathways, other data indicate that miR-9 up-regulates oncogenic pathways in cancer including proliferation, metastasis, and invasion. An investigation of glioma found that inhibition of miR-9 reduced tumor cell stemness and led to cellular differentiation (Schraivogel et al., 2011). It was also shown to increase cell motility, and overexpression down-regulated α-catenin in CRC, indicating involvement in metastasis (Zhu et al., 2012b). In breast cancer, miR-9 expression was up-regulated in higher grade tumors, indicating that it may be involved in proliferation and or metastasis (Krell et al., 2012). Involvement in these pathways is also supported by research in cervical cancer, where up-regulation of miR-9 was associated with increased cellular viability, anchorage-independent growth, and migration *in vitro* (Wilting et al., 2012). In an endometrial cancer cell line, miR-9 was shown to significantly reduce expression of Forkhead box O1 (FOXO1), a transcription factor, while in another endometrial cancer cell line inhibition of this and several other miRNAs resulted in re-expression of FOXO1, cell cycle arrest, and apoptosis (Myatt et al., 2010). Up-regulation of miR-9 in HL results in repression of Hu antigen R [HuR; responsible for destabilizing messenger RNAs (mRNAs) to regulate gene expression] and DICER1 (a major regulator of mRNA, see **Figure 1**), leading to increased cytokine production by HL cells and attraction of normal inflammatory cells, indicating that miR-9 may be involved in large-scale translational regulation as well as inflammation pathways (Li et al., 2011b;Leucci et al., 2012).

There is conflicting evidence regarding the function and expression of this miRNA in AD and cancer, making interpretation of its roles challenging. miR-9 has been found both up- and down-regulated in affected tissue from AD brains and evidence suggests it may play a role in BACE1 regulation. In cancer, there is evidence that miR-9 can up-regulate oncogenic pathways including proliferation, metastasis, and invasion. MiR-9 promotes stemness and prevents differentiation in glioma, increases cell motility and metastasis in CRC, and promotes proliferation and/or metastasis in breast, endometrial, and cervical cancer, and is involved in translational regulation and inflammation pathways in HL.

The evidence suggests that miR-9 may be important in AD, as it has been shown to be differentially expressed in cerebral regions which are significantly associated with pathological progression, yet more research is needed to clarify the molecular causative functions. Cancer research suggests a wide variety of possible functions that could be tested for parallel associations in AD, from transcriptional regulation, proliferation, inflammation, and high-impact translational regulation, to differentiation. Of note, miR-9 appears to have opposite influence on differentiation in neuronal development and glioma. If miR-9 does up-regulate differentiation, as suggested by the study of neuronal development, we might expect this miRNA to be up-regulated in AD, resulting in a decrease in neuronal precursors, and gradual neurodegeneration as a consequence of reduced neuronal renewal. This is supported by the reports of miR-9 up-regulation in the brains of AD patients.

## miR-29

The majority of evidence suggests that miR-29 is down-regulated in AD, though, similar to miR-9, there is some conflicting evidence regarding expression pattern. miR-29 was inversely correlated with the density of amyloid plaques in adjacent tissue in brains from individuals with AD (Wang et al., 2011). miR-29b was also observed to be down-regulated in the parietal lobe cortex of individuals with AD compared to age-matched controls (Nunez-Iglesias et al., 2010). Additionally, loss of miR-29a/b in sporadic AD was correlated with increased BACE1 protein expression, supporting down-regulation of miR-29 in AD and also providing evidence that miR-29 may be directly involved in the amyloid pathway (Hebert et al., 2008). By contrast, in another study miR-29a/b expression was increased in the medial frontal gyrus in AD patients (Cogswell et al., 2008). This finding may reflect region-specific tissue expression; however, it is also important to consider methodological differences in this case. While the three papers documenting down-regulation of this miRNA utilized *in situ* hybridization by microarrays to measure expression,Cogswell et al. (2008) used real-time PCR. This method could introduce additional bias, since it requires primer amplification instead of direct measurement; therefore the consensus of the three other papers on miR-29a/b down-regulation may be more credible in this instance.

miR-29 also has been shown to inhibit neuronal apoptosis during development via inhibition of genes in the pro-apoptotic BH3-only family, which would otherwise inhibit pro-survival proteins in the BCL-2 family (Kole et al., 2011). If one of the primary functions of miR-29 is inhibition of apoptosis, one might expect to observe down-regulation in AD associated with an increased rate of neurodegeneration caused by members of the pro-apoptotic BH3-only gene family inhibiting BCL-2 proteins. For the majority of studies just mentioned, miR-29 was in fact down-regulated in association with disease pathology as predicted by this hypothesis. Most current evidence suggests that miR-29 down-regulation is associated with AD pathology. The up-regulation observed byCogswell et al. (2008) may reflect measurement in the medial frontal gyrus, a region which was not included in other studies.

In cancer, miR-29 appears to interact directly with the BCL-2 family pro-survival proteins. It was down-regulated in malignant cholangiocarcinoma cells with corresponding up-regulation of anti-apoptotic myeloid cell leukemia sequence 1 (MCL-1, a member of the BCL-2 anti-apoptotic family). Transfection of miR-29 decreased MCL-1 levels and increased sensitivity to drug-induced cytotoxicity (Mott et al., 2007). Supporting the validity of these results and suggesting that this function may be common in cancer, miR-29 was observed to be down-regulated in melanoma, mantle cell lymphoma, acute myelogenous leukemia (AML), hepatocellular carcinoma (HCC), cervical cancer, endometrial serous adenocarcinoma, and non-small cell lung cancer (NSCLC), and transfection of miR-29 was correlated with decreased tumorigenicity (Fabbri et al., 2007;Garzon et al., 2009b;Hiroki et al., 2010;Xiong et al., 2010;Zhao et al., 2010;Li et al., 2011d;Nguyen et al., 2011). In AML, miR-29 expression is negatively correlated with MCL-1 mRNA, while in HCC BCL-2 and MCL-1 were both direct targets of miR-29; both of these findings support the theory that miR-29 functions as a tumor suppressor in cancer by promoting apoptosis (Garzon et al., 2009b;Xiong et al., 2010).

In addition to this apoptotic pathway, previous cancer research also contains information linking miR-29 to other pathways. In mantle cell lymphoma, miR-29 was shown to regulate cyclin-dependent kinase 6 (CDK6) mRNA to influence cell division; down-regulation of miR-29 was correlated with increased CDK6 and decreased survival (Zhao et al., 2010). This is supported by research in cervical cancer, which indicated that miR-29 level was negatively correlated with CDK6; addition of miR-29 to human papillomavirus-infected cells was shown to retard cell cycle progression and increase the frequency of apoptosis (Li et al., 2011d). In cholangiocarcinoma cells miR-29 was transcriptionally repressed by NF-κB protein, a key molecule in the inflammatory pathway, as well as c-Myc and hedgehog proteins, implicating this miRNA in proliferation down-regulation (Pikarsky et al., 2004;Mott et al., 2007). In HCC, miR-29 was found to down-regulate matrix metalloproteinase-2 (MMP2) expression, leading to anti-angiogenesis via subsequent suppression of kinase insert domain receptor (KDR, also called VEGFR2) signaling, as well as anti-invasion effects (Fang et al., 2011). Supporting this, in CRC, apolipoprotein B mRNA editing enzyme catalytic polypeptide-like 3G (APOBEC3G) promotes metastasis via inhibition of miR-29 and subsequent up-regulation of MMP2 (Ding et al., 2011). In HCC, miR-29 was also implicated in up-regulation of the long non-coding RNA maternally expressed gene 3 (MEG3), accompanied by inhibited anchorage-dependent and -independent cell growth and increased apoptosis (Braconi et al., 2011). Finally, miR-29 has been associated with transcriptional regulation in cancer via regulation of DNA methylation genes. In melanoma, miR-29 expression was inversely correlated with protein levels of DNA methyltransferases DNMT3A and DNMT3B, which affected overall survival (Nguyen et al., 2011); miR-29 expression was also negatively correlated with DNMT3A and B in lung cancer, and transfection of miR-29 restored normal methylation patterns and expression of various tumor suppressor genes, as well as inhibiting tumorigenicity *in vitro* and *in vivo* (Fabbri et al., 2007). From this evidence, miR-29 appears to act as a tumor suppressor within a large variety of pathways involved in cancer including apoptosis, tumorigenicity, cell cycle regulation, proliferation, invasion, metastasis, angiogenesis, inflammation, and transcriptional regulation.

This analysis of miR-29 implicates involvement in a variety of pathways in cancer, only one of which has been observed in AD. In addition to its activity in the amyloid pathway in AD, down-regulation of miR-29 may allow BH3-only family proteins to inhibit BCL-2 family proteins, leading to apoptosis, while in cancer action of BH3-only family proteins must be blocked somehow, allowing BCL-2 family proteins to promote survival since miR-29 is also down-regulated. The additional interactions of miR-29 in cancer should also be investigated in AD; for example, NF-κB, which is known to be up-regulated in AD, was shown to target miR-29 in cholangiocarcinoma, but to date has not been shown to target this miRNA in AD (Lukiw et al., 2008). Given the findings in cancer, it would be useful to determine whether miR-29 regulates DNA methyltransferases CDK6 and MMP2 in brain, and, if so, whether these interactions influence AD pathology.

## miR-101

Previous studies have demonstrated down-regulation of miR-101 in the temporal and parietal cortex (Hebert et al., 2008;Nunez-Iglesias et al., 2010). Subsequently, miR-101 was shown to down-regulate APP; additionally, lentiviral-mediated overexpression of miR-101 was observed to reduce fibrillar Aβ and another know target, cytochrome *c* oxidase subunit II (COX-2), in hippocampal neurons (Vilardo et al., 2010). Another study replicated miR-101 down-regulation of APP, and also demonstrated that miR-101 is highly expressed in neural cells compared to HeLa and neuroblastoma cells (Long and Lahiri, 2011). These studies indicate that miR-101 down-regulation results in increased Aβ and COX-2, which has previously been shown to induce the innate immune complement component C1qB, and has been associated with inflammation (Seibert et al., 1994;Spielman et al., 2002). Further, COX-2 is elevated in hippocampal tissue in AD cases, and is correlated with amyloid plaque density (Ho et al., 1999). miR-101 therefore may play an important regulatory role in the amyloid, inflammatory, and immune pathways, and down-regulation may exacerbate disease pathology.

There is an extensive body of research on the multiple roles of miR-101 in cancer. miR-101 is consistently down-regulated in many cancers including acute lymphoblastic leukemia, HCC, glioblastoma, lung, gastric, colon, renal, prostate, ovarian, bladder, and pancreatic cancer (Varambally et al., 2008;Strillacci et al., 2009;Su et al., 2009;Hiroki et al., 2010;Smits et al., 2010;Wang et al., 2010a;Banerjee et al., 2011;Hao et al., 2011;Kottakis et al., 2011;Semaan et al., 2011;Thu et al., 2011;Zhang et al., 2011a;Au et al., 2012;Bao et al., 2012;Carvalho et al., 2012;Liu et al., 2012c;Luo et al., 2012). Down-regulation was associated with many oncogenic characteristics including increased proliferation, invasion, advanced tumor stage, and decreased survival; cellular transfection with miR-101 resulted in increased apoptosis and inhibited proliferation, invasion, and angiogenesis, via inhibition of primary target molecules such as the histone methyltransferase enhancer of zeste homolog 2 (EZH2), COX-2, and MCL-1. EZH2 is a transcriptional regulatory molecule which can potentially regulate many different pathways, greatly increasing the impact of miR-101 activity or loss. Redundancy is reflected in miR-29 and miR-101 regulation of the anti-apoptotic MCL-1, indicating that this is an important pathway in cancer and highlighting the potential for future AD research, given that molecules involved in cell survival could play an important role in neurodegeneration. As discussed earlier, COX-2 has been implicated in inflammatory and immune pathways; this functional interaction is seen in both AD and cancer, reflecting the importance of these pathways in both diseases and indicating that miR-101 may be a key negative regulator of both diseases.

## miR-107

miR-107 down-regulation has been reported in AD and has been related to amyloid plaque density, neuritic plaque counts, and neurofibrillary tangle counts in adjacent tissue (Nelson and Wang, 2010;Wang et al., 2011). Additionally, miR-107 has been correlated with BACE1 mRNA level, and BACE1 has been shown to have miR-107 sequence target sites. Thus in AD, miR-107 may be a regulator of BACE1 activity and consequently affect APP cleavage (Wang et al., 2008a;Nelson and Wang, 2010). These results support the role of miR-107 down-regulation in AD and suggest a mechanism related to plaque burden.

Regulation in cancer is more of a mixed picture; there is conflicting evidence for miR-107 regulation in prostate cancer and gastric cancer, indicating that more research on this topic is needed. In prostate cancer, there is evidence that down-regulation of miR-107 is correlated with up-regulation of the mitogen and growth factor granulin (GRN); other members of this miRNA family have also been shown to display this pattern of down-regulation with associated up-regulation of GRN in 11 different types of cancer (Wang et al., 2010b). In contrast, a significantly higher level of miR-107 was found in the urine of men with prostate cancer, suggesting that this miRNA may be up-regulated (Bryant et al., 2012). Additional research is needed to resolve this apparent discrepancy. In gastric cancer, there is also conflicting evidence for regulation of miR-107; expression was significantly elevated in gastric tumor tissue compared to normal tissue, was associated with depth of invasion, lymph node metastasis, and stage, decreased overall survival and disease-free survival, and was inversely correlated with DICER1 mRNA, indicating possible involvement in translational regulation (Li et al., 2011c;Inoue et al., 2012). However, miR-107 was also observed to be silenced in gastric cancer cells, and transfection of miR-107 was correlated with down-regulation of CDK6 mRNA and protein, cell cycle arrest, and decreased proliferation and invasion (Feng et al., 2012). At this time, more research is needed to clarify the roles of miR-107 in prostate and gastric cancer, and whether it is ultimately oncogenic or tumor suppressive.

miR-107 has also been shown to be up-regulated in breast cancer, and down-regulated or silenced in HNSCC, colon cancer, and pancreatic cancer. In breast cancer, miR-107 was found to be up-regulated, was shown to silence the tumor suppressive miRNA let-7, and was associated with increased tumorigenic potential and metastases in mice (Chen et al., 2011). In HNSCC, low expression of miR-107 was correlated with increased protein kinase Cδ (PKCδ), and miR-107 inhibited proliferation, DNA replication, colony formation, and invasion (Datta et al., 2011). miR-107 has been implicated in angiogenesis in colon cancer; the tumor suppressive transcription factor P53 can mediate transcription of miR-107, and forced expression of miR-107 suppresses expression of hypoxia inducible factor-1beta (HIF-1b), suppressing angiogenesis, tumor growth, and vascular endothelial growth factor (VEGF) expression (Yamakuchi et al., 2010). Finally, in pancreatic cancer cell lines, miR-107 was reported to be epigenetically silenced, and re-expression by a demethylating agent in tumor cell lines inhibited CDK6 and decreased growth *in vitro*, indicating that miR-107 is also involved in a cell cycle regulatory pathway (Lee et al., 2009). It is possible that the regulation of let-7 by miR-107 in breast cancer overrides other interactions since let-7 has been shown to affect a large number of targets, and that this level of regulation may be blocked in other cancers, allowing other miR-107 interactions to take precedence and produce tumor suppressive effects.

A number of different pathways appear to be regulated by miR-107 in cancer including proliferation, invasion metastasis, cell cycle regulation, additive translational regulation (mediated by let-7), and angiogenesis. More information on the potential interaction of miR-107 with DICER1, CDK6, HIF-1b, and GRN could further elucidate the molecular pathways involved in AD, as well as the regulatory process of disease initiation and progression. Progranulin (GRN) is a particularly interesting association, given that it has been found to cause one form of frontotemporal lobar degeneration (van Swieten and Heutink, 2008). The altered expression patterns for various types of cancer and AD may indicate that there is another level of regulation that differs across tissues. This in turn may be modulated by other miRNAs, which could alter the methylation status of miR-107 via interaction with histone methyltransferases.

## miR-125b

Small increases in miR-125b have been observed in the hippocampal region of AD brains post-mortem. Because synapsin II mRNA has been identified as a target for this miRNA, it has been postulated that it may be responsible for synapsin protein deficits observed in the brains of patients with AD (Perdahl et al., 1984;Qin et al., 2004;Krek et al., 2005;John et al., 2006;Lukiw, 2007, 2012). miR-125b has been shown to be significantly up-regulated in the temporal lobe neocortex of AD patients (Sethi and Lukiw, 2009;Lukiw et al., 2012). miR-125b was also found to be up-regulated in the hippocampus, medial frontal gyrus, and cerebellum (Cogswell et al., 2008). Further, miR-125b was positively correlated with gray matter neurofibrillary tangles in post-mortem AD patients (Wang et al., 2011). Finally, in cultured human neuronal glial cells, miR-125b has been shown to be up-regulated by NF-κB in response to IL-1β and Aβ42-peptide-induced stress; in this model, miR-125b was also shown to target the mRNA of complement factor-H (CFH), known to be an important suppressor of immune and inflammatory pathways in the brain, and lead to decreased expression (Lukiw et al., 2008;Lukiw and Alexandrov, 2012). From these reports, miR-125b is implicated in neuropathological change, and appears to function in the inflammatory and oxidative stress NF-κB pathways, innate immunity via CFH, and cellular transport via synapsin.

In cancer, miR-125b appears to function mostly as a tumor suppressor. The majority of research to date has been done in HCC; down-regulation of miR-125b has been linked to tumor progression and metastasis, tumorigenicity, proliferation, migration, invasion, angiogenesis, and cell cycle de-regulation in HCC or HCC cell lines (Liang et al., 2010;Alpini et al., 2011;Au et al., 2012). In this disease, miR-125b was shown to be silenced by methylation; miR-125b was also shown to decrease angiogenesis via regulation of placenta growth factor (PIGF), a member of the VEGF family, as well as MMP2 and MMP9, which may be involved in miR-125b effects on invasion (Alpini et al., 2011). Investigation of the effects of miR-125b on angiogenesis in more depth in epithelial cells reveal contradictory evidence that VEGF can induce miR-125b, which in turn inhibits translation of vascular endothelial cadherin (VE-cadherin) and *in vitro* tube formation; overexpression of miR-125b was shown to result in non-functional blood vessel formation, indicating that miR-125b could promote angiogenesis (Muramatsu et al., 2012). It is possible that this contradiction is caused by the model; miR-125b may act through different pathways in cancerous tissue. miR-125b was also shown to up-regulate cyclin-dependent kinase inhibitor 1 (CDKN1A) expression, which arrested cell cycle transition and silenced LIN28B; these effects were linked to inhibited growth, migration, and invasion (Liang et al., 2010). The histone methyltransferase EZH2 was shown to down-regulate miR-125b in HCC, resulting in progression and metastasis (Au et al., 2012). In bladder cancer, miR-125b also appears to function as a tumor suppressor; miR-125b inhibits E2F3, which is involved in the G1/S phase cell transition, and transfection of miR-125b in bladder cancer cell lines decreased E2F3 protein and cyclin A2, depressing colony formation *in vitro* and tumor development in mice (Huang et al., 2011). Contrary to these results, a study in glioma found that miR-125b promotes glioma cell proliferation and inhibits drug-induced apoptosis, indicating that it may play an oncogenic role in this particular disease; the authors further found that miR-125b may target and down-regulate apoptosis-related protein BCL-2 modifying factor, and apoptotic activator (BMF;Xia et al., 2009). Aside from glioma, miR-125b seems to play a tumor suppressive role in a variety of molecular pathways, though miR-125b should be investigated to determine whether this is also true in other cancer types.

Given the largely tumor suppressive function of miR-125b, which inhibits growth and is down-regulated in cancer, we would expect miR-125b to be up-regulated in AD with resulting neurodegeneration, which has been observed as noted above. However, the specific interactions of miR-125b with molecules in a large variety of pathways have not been investigated in AD; research to determine whether miR-125b directly interacts with the molecules listed above including angiogenic VEGF, cell cycle regulatory E2F3, and apoptotic activator BMF could shed more light on the molecular mechanisms involved in AD pathology and further elucidate the pathways involved in this disease. Interestingly, the role of miR-125b in innate immunity does not appear to have been specifically investigated in cancer at this point; this could be an important factor in cancer etiology which should be further investigated as well.

## miR-146

Similarly to miR-125b, miR-146a was found to be significantly up-regulated in the temporal cortices of patients with AD (Sethi and Lukiw, 2009;Lukiw et al., 2012). It was shown to function similarly to miR-125b in the AD inflammatory and oxidative stress pathways, as it was shown to be up-regulated by NF-κB in response to IL-1β and Aβ42 or oxidative stress in cultured human neuronal glial cells, and to decrease expression of CFH (Lukiw et al., 2008;Cui et al., 2010;Lukiw and Alexandrov, 2012). Consistent with these results, miR-146a was observed to be up-regulated in AD neocortices, and in IL-1β and Aβ42 stressed human astroglial cells; in the astroglial model miR-146a down-regulates the Toll-like receptor signaling molecule IL-1 receptor-associated kinase 1 (IRAK1) in tandem with IRAK2 up-regulation by NF-κB to promote the innate immune and inflammatory responses (Cui et al., 2010). Interestingly, unlike miR-146a, miR-146b was down-regulated in both the hippocampus and medial frontal gyrus in individuals with AD; the researchers postulate that down-regulation of miR-146b relieves inhibition of IRAK1 and another TLR signaling molecule, TNF receptor-associated factor 6 (TRAF6), triggering the innate immunity pathway which contributes to the activation of microglia and neurodegeneration (Cogswell et al., 2008). More work is needed to clarify these apparently contradictory roles of miR-146a/b in AD.

Up-regulation of miR-146a was observed in anaplastic thyroid carcinoma (ATC) and cervical cancer, while down-regulation was observed for miR-146a in pancreatic cancer, and miR-146a and b in prostate and breast cancer, indicating that these miRNAs may play a variety of oncogenic or tumor suppressive roles in different cancers (Bhaumik et al., 2008;Lin et al., 2008;Wang et al., 2008b;Hurst et al., 2009;Li et al., 2010;Pacifico et al., 2010;Man et al., 2011). mir-146a has been characterized as oncogenic in ATC, activated by the inflammatory molecule NF-κB; inhibition of miR-146a decreased oncogenic potential in ATC-derived cells and increased susceptibility to chemotherapy-induced cytotoxicity (Pacifico et al., 2010). Additional oncogenic function was observed in cervical cancer, wherein miR-146a was significantly up-regulated in cervical cancer and was tied to cellular proliferation (Wang et al., 2008b). In contrast, miR-146a and b have also been shown to have a variety of tumor suppressive functions in breast, prostate, and pancreatic cancer. Functional interaction research in breast cancer indicates that breast cancer metastasis suppressor 1 (BRMS1) up-regulates miR-146a and b; expression of either miRNA resulted in down-regulation of epidermal growth factor receptor (EGFR) and reduced invasion, migration, and metastasis (Hurst et al., 2009). Also in breast cancer, miR-146a and miR-146b expression suppressed IRAK1 and TRAF6, which was reported to impair NF-κB activity, resulting in reduced invasion and migration (Bhaumik et al., 2008). This negative feedback loop was also observed previously in an acute monocytic leukemia cell line, indicating that miR-146a and b appear to be involved in negatively as well as positively regulating oxidative, immune, and inflammatory responses (Taganov et al., 2006). Transfection of miR-146a in prostate cancer cell lines led to significantly reduced expression of ROCK1 and reduced proliferation, invasion, and metastasis; down-regulation of either miR-146a or b in prostate cancer was also linked to focal basal cell layer disruptions, which have been correlated with invasion (Lin et al., 2008;Man et al., 2011). Similarly, re-expression of miR-146a induced by isoflavone in pancreatic cancer was also correlated with reduced invasion and down-regulation of IRAK1 and EGFR, indicating that this molecular pathway is involved in multiple cancers (Li et al., 2010). Finally, genetic studies have linked polymorphisms in miR-146a to increased risk of gastric cancer, breast cancer, and papillary thyroid carcinoma, while meta-analyses have also linked risk to more generalized cancer susceptibility (Tian et al., 2010;Zeng et al., 2010;Qiu et al., 2011;Lian et al., 2012;Wang et al., 2012a). These various disparate pieces of evidence indicate that miR-146a plays a variety of roles in different cancer types, though there seems to be a commonality to the interactions observed for the tumor suppressive roles miR-146a plays in prostate, pancreatic, and breast cancer. More research would be helpful in clarifying the mediatory mechanisms which determine whether miR-146a is tumor suppressive or oncogenic.

Comparing the evidence for miR-146a function in AD and cancer, there seems to be more overlap than for the miRNAs previously reviewed, though results from cancer may still inform additional interactions in AD. For instance, an investigation of miR-146a regulation of EGFR in AD may tie this miRNA to a proliferative pathway; logically, if these molecules are negatively regulated and miR-146a is up-regulated in AD, one would expect a decrease in EGFR, with possible subsequent neurodegenerative effects. For example, in rodents with traumatic brain injury, treatment with epidermal growth factor reduced hippocampal neuronal cell loss and improved cognitive function (Biessels et al., 2006). Interestingly, modulation of the inflammatory/innate immune pathway by miR-146a appears to be play a large role common to both diseases. The evidence that miR-146a and b inhibit IRAK1 and TRAF6 in breast cancer may contradict the hypothesis ofCogswell et al. (2008) that miR-146b relieves inhibition of these molecules in AD, though a functional study of this interaction, as well as the subsequent NF-κB modulation, would be required for clarification. Finally, it is important that future studies make an effort to clarify the role of miR-146b in cancer, as many results to date have concerned miR-146a; since there seems to be functional overlap based on current research, it would be interesting to investigate further whether these two miRNAs may be differentially regulated in AD.

## miR-153

This miRNA has not been studied as extensively as others mentioned in this review, but is discussed because it has been very recently implicated in regulation of APP.Long et al. (2012) found that delivery of miR-153 in human fetal brain cultures reduced expression of APP, as well as an APP paralog, APLP2, via direct interaction with a target site on the APP 3′ UTR, while inhibition of miR-153 resulted in increased expression of APP; supporting this, decreased miR-153 and increased APP were observed in a subset of AD patients with moderate pathology (Long et al., 2012). Given this important functional evidence for miR-153 interaction in the amyloid pathway, it seems important to review current research of this miRNA in cancer to see if we can extrapolate any further roles or further elucidate molecular function for miR-153.

As with the results for AD, a review of cancer research does not yield much information on miR-153; however, the few studies published may shed additional light of the function of this regulatory molecule. In ovarian cancer, miR-153 was down-regulated, and expression was different for four histopathological types of ovarian cancer; the researchers subsequently found that miR-153 was significantly correlated with tumor grade and clinical stage (Kim et al., 2010). A key study found that miR-153 is down-regulated in glioblastoma, with re-expression in cell lines associated with decreased proliferation and increased apoptosis; the researchers further demonstrated that miR-153 directly inhibited anti-apoptosis molecules BCL-2 and MCL-1 (Xu et al., 2010). An additional study in the same model found that miR-153 also inhibits the insulin receptor substrate 2 (IRS2), further implicating it in growth regulation (Xu et al., 2011). These studies characterize the tumor suppressive role of miR-153, as well as specific interactions which appear to mediate this function. Interestingly, the same endometrial cancer study mentioned previously for miR-9 also found that miR-153 has similar oncogenic properties, appearing to down-regulate FOXO1 and apoptosis (Myatt et al., 2010). This oncogenic function seems to contradict other tumor suppressive functions listed above and indicates that this miRNA would benefit from more intense scrutiny.

Mediation of BCL-2 and MCL-1 by miR-153 indicates that it may have overlapping functionality with miR-29 and 101; however, the majority of evidence indicates that these miRNAs are down-regulated in AD, whereas if they were inhibiting anti-apoptotic molecules, we would expect them to be up-regulated in AD leading to neurodegeneration. Additional research is needed to determine if miR-153 can also inhibit anti-apoptosis molecules in the brain, as well as why this interaction would be suppressed in a neurodegenerative disease like AD. Interestingly, if miR-153 does in fact interact with IRS2, that would tie this miRNA to the insulin regulatory pathway; in addition to the known association of diabetes with increased dementia risk, there is some evidence that insulin may be directly involved in AD, so this association should be a focus of further investigation (Watson and Craft, 2003;Biessels and Kappelle, 2005;Craft, 2007;Whitmer, 2007).

## miR-195

There is comparatively little evidence for the involvement of miR-195 in AD; however, miR-195 has been shown to directly target the 3′ UTR ofBACE1, and to decrease protein expression in mouse neuroblastoma cells, with a corresponding drop in Aβ levels (Zhu et al., 2012a). Additionally, miR-195 was decreased in the cerebral spinal fluid (CSF) of AD patients compared with controls (Cogswell et al., 2008), while another group found that miR-195 was down-regulated and was negatively correlated with diffuse amyloid plaques in the gray matter of post-mortem AD patients (Wang et al., 2011). Given these limited but interesting results, it seems important to review the roles of miR-195 in cancer to see if additional information about miR-195 in the brain can be extrapolated for further investigation. Since down-regulation could theoretically be tied to increased amyloid beta levels, establishing expression patterns of this miRNA in AD brain tissue should be a research priority, along with more functional investigation.

Importantly, miR-195 has been extensively studied in cancer, with current research indicating that it plays tumor suppressive or oncogenic roles in different cancer types. Down-regulation was observed in a number of different cancer types, and was associated with poorer overall survival, increased metastasis incidence, and higher tumor stage; however, up-regulation was also observed in several cancer types (Zanette et al., 2007;Flavin et al., 2009;Guled et al., 2009;Soon et al., 2009;Xu et al., 2009;Liu et al., 2010;Moser and Fritzler, 2010;Ozata et al., 2011;Guo et al., 2012;Lin et al., 2012;Wang et al., 2012b;Zhang et al., 2012). In breast cancer and glioma, conflicting studies indicate that more research is needed to determine the impact of this miRNA (Heneghan et al., 2010a,b;Ujifuku et al., 2010;Lakomy et al., 2011;Li et al., 2011a). Research in the tumor suppressive functions of miR-195 has shown that it interacts with a number of molecules involved in cell cycle regulation, as well as metabolic and anti-apoptotic molecules. miR-195 was shown to inhibit cyclin D1 in HCC and breast cancer (Xu et al., 2009;Li et al., 2011a), CCND3 in glioblastoma (Zhang et al., 2012), CDK4 in bladder cancer (Lin et al., 2012), CDK6 in HCC (Xu et al., 2009), E2F3 in HCC and glioblastoma (Xu et al., 2009;Zhang et al., 2012), BCL-2 in breast and colorectal cancer (Liu et al., 2010;Singh and Saini, 2012), RAF1 in breast cancer (Li et al., 2011a), and GLUT3 in bladder cancer (Fei et al., 2012). The role of miR-195 in suppression of anti-apoptotic BCL-2 lends support to the evidence that miR-195 is down-regulated in breast cancer, promoting cell survival. Unsurprisingly given these genes’ known roles in cell cycle regulation, metabolism, and anti-apoptosis, inhibition via miR-195 was variously associated with cell cycle arrest, decreased proliferation, reduced invasion, increased apoptosis, and reduced colony formation and tumor formation. Interestingly, given that the only clinical measure associated with up-regulation at this point is increased resistance to temozolomide (Ujifuku et al., 2010), it is possible that the tumor suppressive functions of miR-195 early in cancer development are overridden by oncogenic interactions favoring survival in the context of drug resistance; further investigation of this is merited in cancers associated with miR-195 up-regulation as listed above. The dearth of functional and clinical investigation of miR-195 up-regulation, as well as the contradiction between research indicating that it may be up or down-regulated in breast cancer and glioblastoma, should be addressed to further clarify the mechanisms of miR-195 in cancer.

From the current literature detailing the tumor suppressive role of miR-195 in various cancers, it is possible to hypothesize that this miRNA has functional overlap with miR-29 and miR-153, both of which have been shown to interact with BCL-2, which would lead to the additional hypothesis that miR-195 is similarly down-regulated in AD. This is a logical expectation given the functional evidence that decreases in miR-195 could be tied to increased amyloid beta and decreased levels were observed in CSF and gray matter, however, it requires further investigation. Researching these premises might also point to the possibility that all three miRNAs are regulated via a common mechanism in AD given their apparent redundancy. As suggested earlier, further investigation in AD of the functional interactions ascribed to this miRNA in cancer might help elucidate additional mechanisms by which it influences AD pathology. Given the evidence in cancer, it is possible that this miRNA is also important in proliferative pathways in the brain.

## PATHWAYS

**FIGURE 4:**
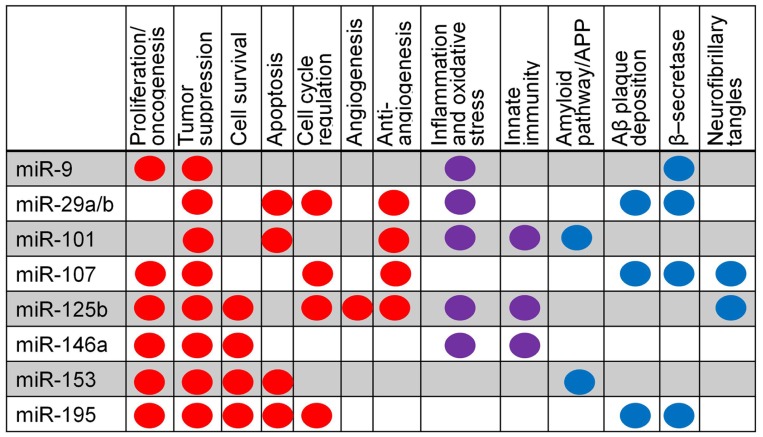
**Summary of miRNA pathway relationships in cancer and AD.** Major pathways are listed; dots indicate evidence that a specific miRNA is involved in a particular pathway. Red indicates evidence from cancer research, blue from AD research, and purple from both cancer and AD.

The overall involvement of miRNAs under consideration in this review in specific biological pathways is summarized in **Figure 4**, including oncogenic and tumor suppressive functions in cancer and amyloid-specific mechanisms. The amyloid pathway has clearly been a focus of AD research to date. We have reviewed evidence that multiple miRNAs regulate expression of molecules in this pathway; mir-9, -29a/b, -107, and -195 have been predicted or shown to target, or negatively correlated with, BACE1 mRNA or protein levels, suggesting that these miRNAs may have functional overlap or redundancy, while miR-101 and -153 have been shown to inhibit APP protein directly (**Figure 2**).

In addition, miR-9, 125b, and 146a are up-regulated by the inflammatory and oxidative stress responses, suggesting that the amyloid pathway may have wide-reaching regulatory impact via miRNA.

**FIGURE 5:**
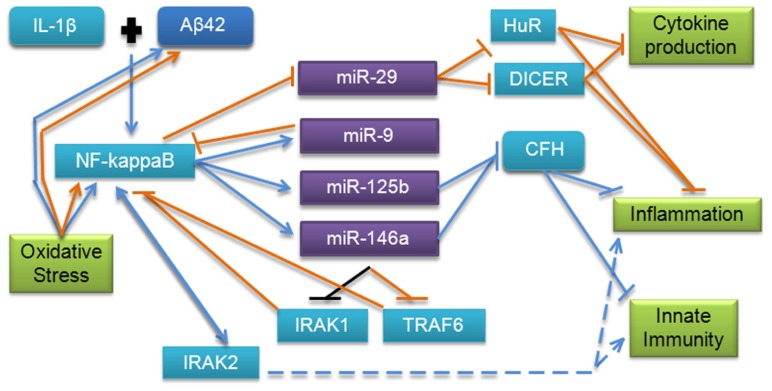
**MicroRNA involvement in the inflammation, innate immunity, and oxidative stress pathways in AD and cancer**. This pathway has been validated by several studies in AD; however, additional evidence from cancer research points to more molecules which may be involved. Blue lines indicate interactions and effects found in AD research, orange lines indicate interactions and effects found in cancer research, and black lines indicate findings common to both diseases. Dashed lines indicate downstream effects.

The inflammatory and innate immune pathways have been extensively investigated in both cancer and AD. miRNAs appear to be important in these pathways in both diseases and may function using some of the same mechanisms (**Figure 5**). Regulation involving the major inflammatory and oxidative stress transcription factor NF-κB is a common theme observed for many of the miRNAs reviewed here. This molecule is a key signaling molecule in AD as it appears to tie together the amyloid, inflammatory, oxidative stress, and innate immunity pathways, but it has also been shown to play an important role in cancer. As discussed earlier in this review, in cancer, miR-9 down-regulates NF-κB and NF-κB appears to directly down-regulate miR-29a and up-regulate miR-146a, while in AD, NF-κB appears to up-regulate miR-9, miR-125b, and miR-146a. miR-125b and -146a regulate several other molecules in the NF-κB pathway and indirectly modulate NF-κB; miR-146a has been shown to down-regulate TLR signaling molecules IRAK1 and TRAF6 in cancer, with a corresponding drop in NF-κB level. Interestingly, miR-146a was also shown to down-regulate IRAK1 in AD, but did not affect TRAF6 in the Aβ42 and IL-1β-stressed human cultured astroglial cell model (Cui et al., 2010). This process has been postulated to result in “fine-tuning” of these pathways, and may be aided by miR-9 down-regulation of NF-κB, though this remains to be verified in AD. Given the similar expression and activation of miR-125b compared with miR-146a, it would also be interesting to investigate whether this miRNA may also regulate downstream TLR signaling via a similar mechanism.

Additionally, in AD, mir-125b and -146a have been shown to regulate CFH, which normally represses the cerebral inflammation response. CFH has also been shown to play an important regulatory role in the complement cascade, which is a major component of innate immunity. Dysregulation has also been observed in Down’s syndrome, which shares some neuropathological characteristics of AD (Li et al., 2012). Interestingly, *CFH* polymorphisms have been previously associated with increased risk of AD in apolipoprotein E ε4 carriers, indicating that this miRNA regulatory mechanism could be an important factor in pathology, and should be further investigated (Zetterberg et al., 2008). Based on the evidence in cancer research, an investigation of the interaction of miR-9 and -29b with NF-κB in AD and possible downstream targets in these pathways may further clarify the molecular mechanisms involved. mir-101 regulation of COX-2, which has been associated with innate immunity and inflammation, adds complexity to this regulatory pathway. We conclude that the innate immune, oxidative stress, and inflammatory pathways regulated by miRNAs are important in cancer and AD, and utilize some overlapping functional mechanisms; further research into each area based on previous research presented here could help to advance knowledge in both disease areas.

To date, most miRNA research on functional molecular interactions in AD has focused on the amyloid and inflammation pathways since these have been directly implicated in neuropathology. However, a number of other pathways have been postulated or demonstrated to play a role in AD including proliferation and angiogenesis. Metastasis and invasion, key topics in cancer research, have been shown to be effectively regulated by miRNAs.

**FIGURE 6:**
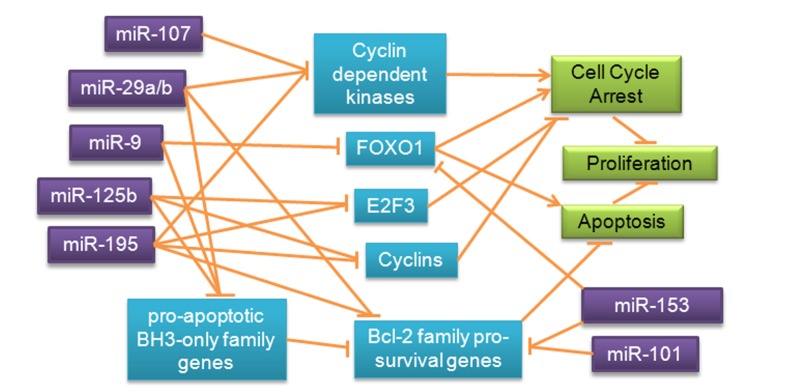
**Redundant miRNA mechanisms regulating proliferation and survival pathways in cancer**. Expression of transcription factors, BCL-2 family genes and inhibitors, and cell cycle regulatory molecules is redundantly regulated by miRNAs in cancer, prompting speculation about possible regulatory roles of these miRNAs in a similar pathway in AD.

Given that some of the underlying functions of extracellular matrix degradation, stemness, and differentiation could affect neurophysiology and be involved in AD, miRNA regulation of matrix metalloproteinases, cyclin-dependent kinases, and cyclins, all of which have been observed in cancer, should also be investigated in AD. Proliferative molecules including GLUT3 and IRS2, which are specifically involved in cell metabolism and appear to be differentially regulated by miRNAs in cancer, could also contribute to risk for AD and increased rates of neurodegeneration via impaired metabolism. Pro-survival genes such as BCL-2 and MCL-1 have been observed to be redundantly regulated by multiple miRNAs in cancer; inappropriate silencing of these molecules by miRNAs in AD could exacerbate neurodegeneration. Cell cycle regulatory molecules such as cyclin-dependent kinases, cyclins, and E2F3, are shown to be targets of several of the miRNAs discussed, indicating that these miRNAs can inhibit the cell cycle (**Figure 6**).

Amyloid beta appears to up-regulate angiogenesis, suggesting that this may be an important area for future miRNA research in AD (Cameron et al., 2012). As discussed earlier, various miRNAs have been shown to play important roles in angiogenesis in cancer. miR-29 down-regulates MMP2, indirectly reducing VEGFR2 signaling and angiogenesis, miR-107 reduces HIF-1b, with subsequently reduced angiogenesis, tumor growth, and VEGF expression, and miR-125b was shown to inhibit PIGF and VE-cadherin in different cell lines, inhibiting angiogenesis.

Interestingly, all instances of angiogenic regulation observed here were tumor suppressive, indicating a degree of functional redundancy, but one of the four miRNAs implicated was up-regulated in AD. miR-125b may be differentially expressed because it has been shown to be up-regulated by amyloid beta. TRAF6, the innate immune molecule down-regulated by miR-146a in breast cancer, has also been shown to negatively regulate VEGF in epithelial cells, leading to the question of whether the miR-146a regulatory pathway may result in increased VEGF signaling and angiogenesis (Bruneau et al., 2012). This pathway, which has been implicated in AD, should be further investigated for miRNA regulation to elucidate function and disease risk factors.

Transcriptional and translational regulation of miRNAs and target genes is also a key process in both diseases, with multiple hierarchical levels of control and feedback mechanisms playing important roles in effect size and specificity. While miRNAs are inherently involved in translational regulation via the RISC silencing complex (**Figure 1**), several miRNAs in this review have may affect translational regulation at a higher level; both miR-9 and -107 have been shown to inhibit DICER1, and miR-107 has been shown to inhibit another miRNA, let-7. Interestingly, miR-29 and -101 have been implicated in transcription control via inhibition of methyltransferases. An investigation of these properties could elucidate additional functions of these miRNAs and provide information on this important regulatory pathway in AD.

## CONCLUSION

MicroRNAs are clearly a relevant topic for both cancer and neurodegenerative disease. Many of the miRNAs discussed in this review have differential expression in AD and cancer, suggesting that they play multiple regulatory roles in pathways active across both cancer and AD (**Table 1**). While miRNA has been actively researched in cancer for a number of years, this is a relatively new area for research in AD and other neurodegenerative diseases. From a review of the literature on miRNAs in AD, much work remains to be accomplished to elucidate all roles of miRNAs in pathological pathways. Reviewing a number of miRNAs with functional evidence of involvement in AD indicates that other than the amyloid pathway and inflammation, possible regulation of other pathways by these molecules has been largely ignored. This review of miRNA regulatory mechanisms in cancer and AD has covered a variety of important mole cular mechanisms observed in cancer that could potentially also contribute to pathology in AD. Further investigation of these pathways and mechanisms appears warranted and will hopefully be a focus of future research. Studies addressing other biological pathways such as proliferation and angiogenesis would be important and help fill the gaps in our understanding of the regulatory mechanisms influencing this very complex disease.

**Table 1:** miRNA expression in cancer and AD.

miRNA	Types of Cancer (see text for citations)		Alzheimer’s disease affected tissue	
	Down-regulated	Up-regulated	Down-regulated	Up-regulated
miR-9	Melanoma and HNSCC	Glioma, GC, biliary, Hodgkin lymphoma, CRC, BC, cervical	Hippocampus, medial frontal gyrus, ATC	Hippocampus, TL
miR-29	Cholangiocarcinoma, melanoma, MCL, AML, HCC, cervical, non-small cell lung		SMTG, parietal lobe cortex, ATC	Medial frontal gyrus
miR-101	ALL, HCC, glioma, lung, GC, colon, renal, PC, ovarian, bladder, pancreatic		Parietal lobe cortex, ATC	
miR-107	GC, PC, HNSCC, colon, pancreatic	GC, PC, BC	SMTG (replicated)	
miR-125b	HCC, bladder	Glioma		TL, hippocampus, medial frontal gyrus, cerebellum, HNG
miR-146a	Anaplastic thyroid, cervical	Pancreatic, PC, BC		TL, HNG, human astroglial cells
miR-153	Ovarian, glioma	Endometrial	Frontal cortex	
miR-195	BC, glioma, CRC, bladder, adrenocortical, HCC, primary peritoneal	BC, glioma, astrocytoma, CLL, malignant mesothelioma	CSF, SMTG	

*MiRNAs are significantly differentially expressed, indicating different roles in disease. HNSCC, head and neck squamous cell carcinoma; GC, gastric cancer; CRC, colorectal cancer; BC, breast cancer; ATC, anterior temporal cortex; TL, temporal lobe neocortex; MCL, mantle cell lymphoma; AML, acute myelogenous leukemia; HCC, hepatocellular carcinoma; SMTG, superior and middle temporal gyri; ALL, acute lymphoblastic leukemia; PC, prostate cancer; HNG, human neuronal-glial cells in culture; CLL, chronic lymphocytic leukemia.*

A comparison of research on miRNAs in cancer and AD highlighted areas which could benefit from future research in both diseases. Though the primary focus was on increasing knowledge of AD from previous research in cancer; this case study also demonstrates that a similar strategy could be useful in studying other neurodegenerative diseases, which are starting to experience increasing attention with regard to the roles of miRNAs in disease pathophysiology.

## Conflict of Interest Statement

The authors declare that the research was conducted in the absence of any commercial or financial relationships that could be construed as a potential conflict of interest.

## Acknowledgments

This study was supported in part by the US National Institutes of Health (National Cancer Institute (R01 CA101318, R01 CA087845, P30 CA082709, and R25 CA117865) and the National Institute on Aging (R01 AG19771 and P30 AG10133).
